# Similar Safety Profile of the Enantiomeric N-Aminoalkyl Derivatives of *Trans*-2-Aminocyclohexan-1-ol Demonstrating Anticonvulsant Activity

**DOI:** 10.3390/molecules24132505

**Published:** 2019-07-09

**Authors:** Karolina Słoczyńska, Paulina Koczurkiewicz, Kamil Piska, Beata Powroźnik, Katarzyna Wójcik-Pszczoła, Katarzyna Klaś, Magdalena Wyszkowska-Kolatko, Elżbieta Pękala

**Affiliations:** Department of Pharmaceutical Biochemistry, Faculty of Pharmacy, Jagiellonian University Medical College, 9 Medyczna Street, 30-688 Krakow, Poland

**Keywords:** aminocyclohexanol, cytotoxicity, enantiomers, epilepsy, mutagenicity, P-glycoprotein

## Abstract

Epilepsy is one of the most common neurological disorder in the world. Many antiepileptic drugs cause multiple adverse effects. Moreover, multidrug resistance is a serious problem in epilepsy treatment. In the present study we evaluated the safety profile of three (**1**–**3**) new chiral *N*-aminoalkyl derivatives of *trans*-2-aminocyclohexan-1-ol demonstrating anticonvulsant activity. Our aim was also to determine differences between the enantiomeric compounds with respect to their safety profile. The results of the study indicated that compounds **1**–**3** are non-cytotoxic for astrocytes, although they exhibit cytotoxic activity against human glioblastoma cells. Moreover, **1**–**3** did not affect the viability of HepG2 cells and did not produce adducts with glutathione. Compounds **1**–**3** demonstrated no mutagenic activity either in the *Salmonella typhimurium* or in *Vibrio harveyi* tests. Additionally, the compounds displayed a strong or moderate antimutagenic effect. Finally, the P-glycoprotein (P-gp) ATPase assay demonstrated that both enantiomers are potent P-gp inhibitors. To sum up, our results indicate that the newly synthesized derivatives may be considered promising candidates for further research on anticonvulsant drug discovery and development. Our study indicated the similar safety profile of the enantiomeric *N*-aminoalkyl derivatives of *trans*-2-aminocyclohexan-1-ol, although in the previous studies both enantiomers differ in their biotransformation pathways and pharmacological activity.

## 1. Introduction

Epilepsy is one of the most common neurological disorder in the world. Despite advanced studies on antiepileptic therapies, there is still a lack of effective drugs that are appropriate for patients. The most important challenges in epilepsy treatment include the multiple and serious adverse effects of the currently available antiepileptic drugs (AEDs) and multidrug resistance (MDR) [1,2,3,4,5].

**Figure 1:**
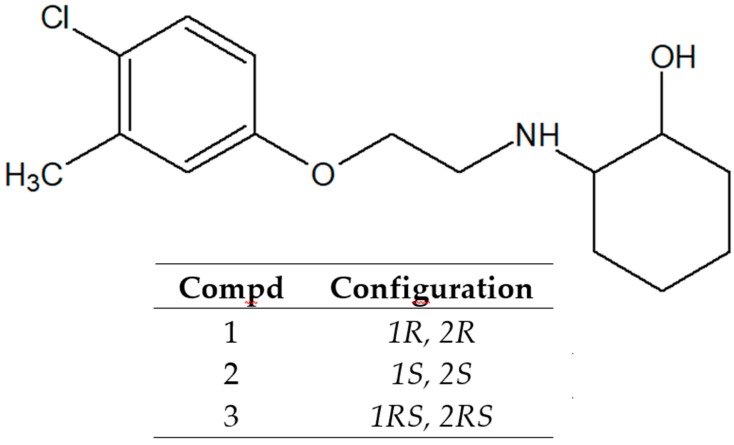
Chemical structures of the title compounds (**1**–**3**).

In the search for new agents that possess antiepileptic activity, we turned our attention to aminocyclohexanol derivatives that exhibit potent central nervous system (CNS) activity [6,7,8]. In the previous study we described the synthesis, in vivo anticonvulsant activity, and in vitro metabolism of three new chiral *N*-aminoalkyl derivatives of *trans*-2-aminocyclohexan-1-ol, denoted as **1** (*R* enantiomer), **2** (*S* enantiomer), and **3** (racemate) (Figure 1) [9]. These compounds were proved to be active anticonvulsants in a maximal electroshock test (MES) performed at the National Institute of Health (Rockville, USA) within the Antiepileptic Drug Development Program [10]. In particular, *R* enantiomer exhibited anti-MES activity with a protective index (PI) of 1.72 (mice, i.p.), whereas racemate showed PI value of 2.83 (mice, i.p.) (Table 1) [9]. Moreover, biotransformation analysis of compounds **1**–**3** performed in the liver microsome system demonstrated that enantiomers *R* and *S* undergo different biotransformation pathways, which could explain their therapeutic potential and toxicity [9].

In light of the aforementioned findings and continuing our study of three active chiral *N*-aminoalkyl derivatives of *trans*-2-aminocyclohexan-1-ol, the present work was focused on in vitro cytotoxicity evaluation and understanding the basic mechanisms that underlie compounds **1**–**3** activity. In particular, we established the safety profile of **1**–**3** by performing cellular cytotoxicity analysis using CNS cells and mutagenicity assays with the bacterial strains *Salmonella typhimurium* and *Vibrio* *harveyi*. As many AEDs are known to demonstrate hepatotoxic effects, we also evaluated the hepatotoxicity of compounds **1**–**3**, both in a HepG2 cellular model and in a human liver microsome system in the presence of glutathione. Additionally, the antimutagenic effects of test substances were determined.

MDR has been described in many patients receiving anticonvulsant treatment. One frequently described mechanism of this phenomenon is overexpression of ATP-binding cassette (ABC) transporters at the blood–brain barrier. High ABC transporter expression is largely responsible for the fact that anticonvulsant drugs are pumped out of their target site of action [11]. P-glycoprotein (*P*-*gp*) *plays a crucial role* in drug absorption and brain uptake, and many studies have demonstrated that several AEDs could be P-gp substrates or inhibitors [5,12,13,14,15]. Therefore, within the present paper, the ability of compounds **1**–**3** to modulate P-gp ATPase activity was assessed to clarify the interactions between the investigated compounds and P-gp and to evaluate their potential as new drug candidates for treating MDR epilepsy.

Finally, our aim was to determine differences between the enantiomeric *N*-aminoalkyl derivatives of *trans*-2-aminocyclohexan-1-ol with respect to their safety profile. 

## 2. Results and Discussion

### 2.1. Cytotoxicity

AEDs are fraught with numerous undesirable effects, of which cytotoxicity is one of the most serious [16,17,18,19,20]. Therefore, in this work we focused our attention on assessing the active compounds’ (**1**–**3**) cytotoxicity in relation to CNS cells. A typical model for neurotoxicity evaluation commonly used in publications are human neuroblastoma SH-SY5Y cells. Considering the fact that the cells of this line are transformed, they have a cancerous origin. Therefore, within this work we decided to investigate the safety profile of compounds **1**–**3** on a normal cell line i.e., astrocytes. Astrocytes are specialized glial cells that contiguously tile the entire central nervous system and play a crucial role in CNS [21]. Interestingly, astrocytes are also vulnerable to seizures, as shown by Gualtieri et al. [22] and reviewed in Curia et al. [23]. Moreover, astrocytes have been used successfully in safety profile analysis of different compounds [24,25,26,27].

**Figure 2:**
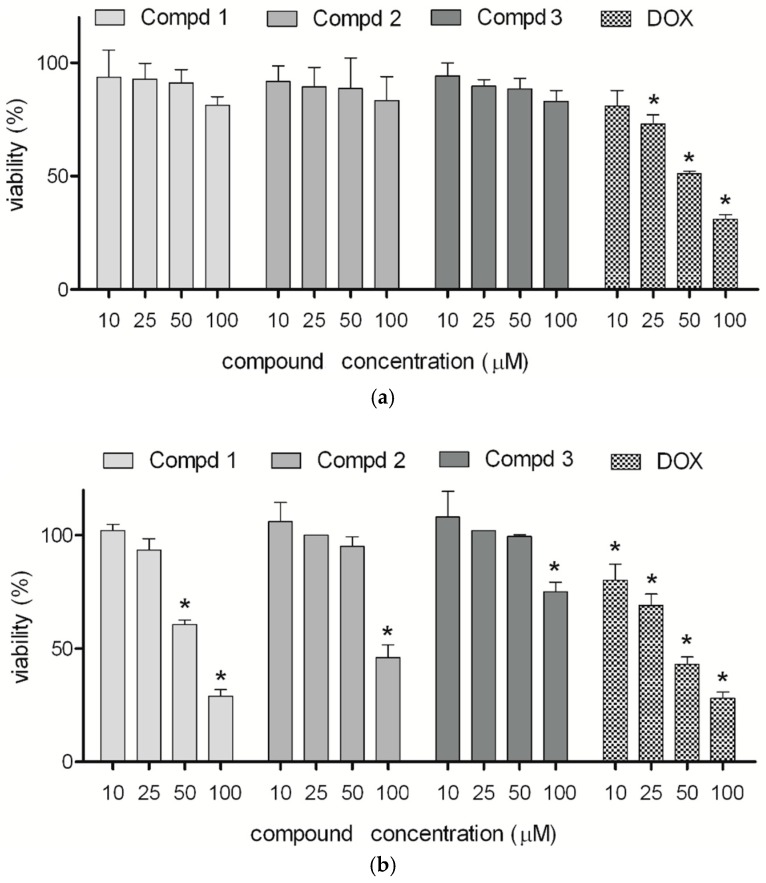
Effect of compounds **1**, **2**, and **3** on viability of astrocytes (**a**) and human glioma cells (**b**). Cells were cultured in medium supplemented with 10% FBS and antibiotics in the presence or absence (control) of compounds (10, 25, 50, or 100 μM). After 24 h incubation, the MTT assay was performed. Doxorubicin (DOX) was used as a positive control. Results from the MTT assay are presented as percent of living cells compared to the control. Values represent means ± SEM of three independent experiments (*p* < 0.05).

**Figure 3:**
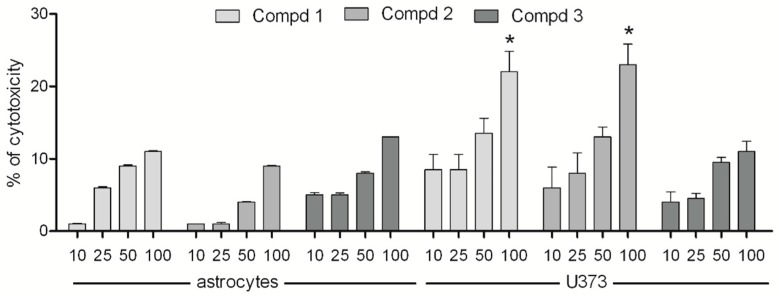
Cytotoxicity of compounds **1**, **2**, and **3** on astrocytes and human glioma cells. Cells were cultured in medium supplemented with 10% FBS and antibiotics in the presence of compounds (10, 25, 50, or 100 μM). Results from LDH assays show the percent of cytotoxicity compared to the control. Values represent means ± SEM of three independent experiments (*p* < 0.05).

The results obtained using the MTT assay demonstrated that none of the tested compounds induced a significant cytotoxic effect on the normal astrocyte cell line in a concentration range of 10–100 µM, while glioblastoma cells incubated with higher concentrations of both enantiomers lose viability in a dose-dependent manner (Figure 2). The racemic compound was less cytotoxic than the two enantiomers. Moreover, the results of the MTT test are comparable with those obtained in the LDH assay (Figure 3). Matysiak et al., who examined the cytotoxicity of (*R*)- and (*S*)-ricinoleic acid amides and their acetates, revealed that both enantiomers of the ethanolamine-derived amides showed promising anticancer potential [28]. On the other hand, in the study of praziquantel enantiomers it was indicated that both enantiomers differ in their cytotoxicity profile [29]. Similarly, according to Tokunaga et al. the enantiomeric forms of fluoro-thalidomide displayed different anti-tumor activities, with the (*S*)-enantiomer being noticeably more potent [30].

The observed selectivity of **1**–**3** towards different cell lines is particularly interesting and opens up new prospects for research on their activity. Cytotoxic activity of tested *trans*-2-aminocyclohexan-1-ol derivatives with chiral carbon atoms against tumor cells may be vital in the context of their potential chemopreventive action. The anti-cancer activity of anti-epileptic drugs has previously been described in the literature; however, there have been no reports concerning compound selectivity. Interestingly, valproic acid prodrug was used to increase the sensitivity of neoplastic cells to doxorubicin therapy [31]. In another study, Kwiecińska et al. demonstrated that co-treatment with valproic acid increased the efficiency of doxorubicin, carboplatin, and cyclophosphamide in various ovarian cancer cell lines [32].

### 2.2. Hepatotoxicity, Metabolic Activation and Trapping Assay

**Figure 4:**
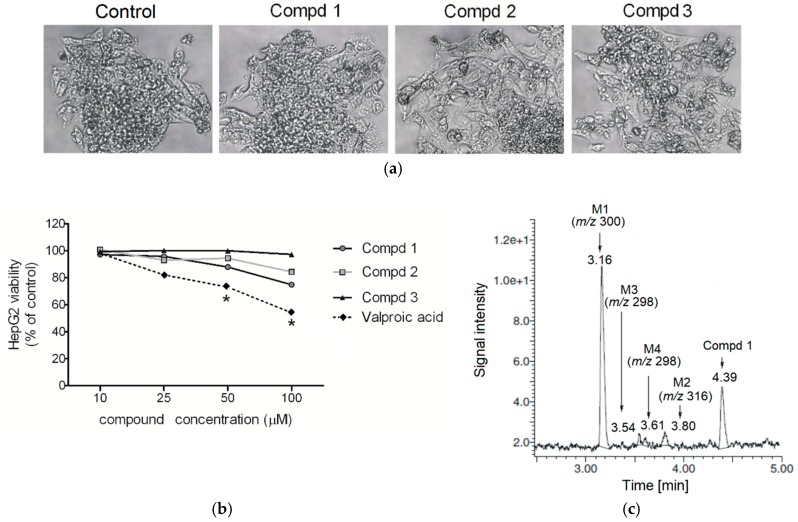
Hepatotoxicity of **1**, **2**, and **3**. Cells were cultured in medium supplemented with 10% FBS in the absence or presence of compounds (10, 25, 50, or 100 μM). After 24 h incubation, pictures of cells were taken using bright field microscope (**a**) and the MTT assay (**b**) was performed. Results from the MTT assay show the percent of living (metabolically active) cells. Values represent means ± SEM of three independent experiments (*p* < 0.05). Valproic acid was used as positive control. Metabolic activation and trapping assay (**c**); representative chromatogram of **1** and its metabolites following incubation of **1** with human liver microsomes supplemented with NADPH and reduced glutathione. Molecular masses of the [M + H]^+^ ions of metabolites are shown.

Drug-induced liver injury is the most common cause of liver injury and a serious clinical problem. Therefore, within the present paper compounds **1**–**3** were evaluated in vitro for their hepatotoxicity on human liver cancer cells (HepG2) in the MTT viability assay. As shown in Figure 4, the tested compounds did not affect the viability of HepG2 cells at concentrations up to 100 µM. Moreover, they did not affect the cellular morphology after 24 h incubation, even at the highest concentration used in the experiment (Figure 4).

Many xenobiotics are known to display hepatotoxic activity, resulting not only from their direct action, but also from their biotransformation to reactive intermediates [33,34]. Such properties are characteristic of classical AEDs, such as valproic acid, which is biotransformed to a toxic metabolite 4-ene-valproic acid. The hepatotoxicity of 4-ene-valproic acid contributes to the development of liver injury [35]. Electrophiles that occur during metabolism form conjugates with intracellular macromolecules and constituents of the antioxidant system (primarily with glutathione); this causes its depletion and contributes to hepatocytes necrosis. For these reasons, early drug development processes include strategies for the detection of reactive metabolites by means of trapping assays with glutathione [36,37,38].

**Figure 5:**
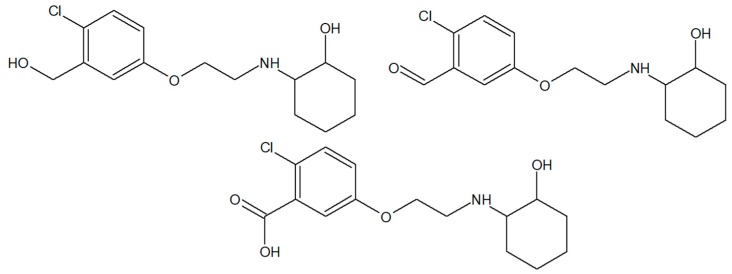
Chemical structures of the main metabolites of compounds **1**–**3**.

Compounds **1**–**3** were examined for the production of toxic metabolites in order to predict their hepatotoxic potential. Incubations of test compounds with glutathione gave several peaks that were attributed entirely to the remaining parent compound. The main metabolites were the products of aliphatic hydroxylation (*m/z* 300) and carbonylation (*m/z* 298 and *m/z* 316) (Figure 5). The representative chromatogram of *R* enantiomer and its metabolites following incubation with human liver microsomes (HLMs) supplemented with NADPH and glutathione is presented in Figure 4. As compounds **1**–**3** did not produce any adducts with glutathione in the human liver microsome system, probably they can be classified as safe with respect to their metabolic fate. These results are consistent with previous reports relating to in vitro biotransformation of compounds **1**–**3** with rat liver microsomes [9].

### 2.3. Mutagenicity and Antimutagenicity

Mutagenic activity is one of the most important endpoints for risk assessment of chemical compounds including both drug substances and drug candidates. Mutagenic agents are capable of inducing unfavorable effects on genetic material of a cell [39]. As regards chiral compounds, enantiomers may possess different carcinogenicity and teratogenicity [40]. On the other hand, the mutagenic properties of some chemicals may be partly modulated or reduced by using agents possessing antimutagenic activity [41].

In the present paper, mutagenicity and antimutagenicity of **1**–**3** were evaluated using two microbial systems i.e., *Salmonella typhimurium* and *Vibrio harveyi*. According to our findings, the racemic compound and both enantiomers demonstrated no mutagenic activity either in the TA100 strain of *S. typhimurium* or in the strains of *V. harveyi* (BB7, BB7M, BB7X, and BB7XM) (Table 2 and Table 3). Additionally, the investigated compounds displayed a strong or moderate antimutagenic effect on chemically induced mutagenesis in *S. typhimurium* and *V. harveyi* strains (Table 2 and Table 3). To determine the antimutagenic activity, a direct mutagen nitroquinoline-N-oxide (NQNO) was used. NQNO is a base substitution agent, principally acting on G residues, inducing mainly GC to AT transitions [42]. The observed protection of the bacterial genome against NQNO damage may be a result of the inhibition of the interaction between DNA and a mutagen [41,43]. However, more studies are needed to confirm this hypothesis.

### 2.4. P-gp Activity

An important approach in the evaluation of **1**–**3** was to determine their interaction with P-gp, which is an integral plasma membrane protein and functions as an ATP-dependent drug efflux pump [44,45]. It was demonstrated that P-gp is involved in MDR and some adverse drug–drug interactions [46,47]. P-gp-interacting pharmaceutical agents are recognized as stimulators or inhibitors of ATPase activity.

**Figure 6:**
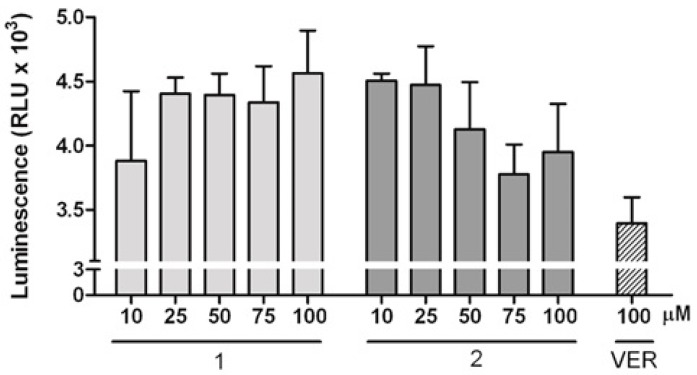
Effects of **1** or **2** and verapamil (VER) on P-gp activity. P-gp ATPase activity was determined by using the P-gp-Glo TM assay system, and ATP accumulation was measured as luminescence in a cellular system in the absence and in the presence of 100 µM verapamil (used as positive control for ATPase activity inhibition) or **1** or **2** at increasing concentrations (10, 25, 50, 75, or 100 µM).

In the present experiment, verapamil (VER), which is an inhibitor as well as a substrate of P-gp, was used as a positive control that stimulates P-gp ATPase activity (ATP consumption) [44]. In the presence of VER a P-gp-dependent decrease in luminescence was observed, whereas in the case of two enantiomers, an increase in luminescence was detected, which indicates a decrease in ATPase activity and, thus, inhibition of the P-gp ATPase function (Figure 6). Therefore, it was demonstrated that both enantiomers possess P-gp inhibition ability, with *R* enantiomer being more potent. It was demonstrated previously that P-gp can interact in a different way with enantiomers [48,49,50]. When chiral drugs modulate P-gp, one enantiomer can increase the activity of the pump, while the other enantiomer can inhibit its activity [51,52].

In general, P-gp inhibition is related to three mechanisms: drug binding site blocking, influence on ATP hydrolysis, and modification of membrane lipid integrity [15]. Interestingly, such inhibition of P-gp may represent an approach to improving treatment efficiency, especially in the field of oncology or pharmacoresistant epilepsy [14,53,54,55,56,57,58]. As regards both tested enantiomers, further studies are definitely needed in order to better understand their P-gp inhibition potential and possible MDR modulation potency.

## 3. Materials and Methods

### 3.1. Cytotoxicity Assays

#### 3.1.1. Cell Culture

Astrocyte cell line (ATCC, CRL-2541), human glioblastoma astrocytoma cell line U373 (ATCC, HTB17-1055), and human hepatoma HepG2 cells (ATCC, HB-8065) were used in the study. The cells were cultured in standard conditions (37 °C, 5% CO_2_), in EMEM medium with 2 mM glutamine and 1% non-essential amino acids, all supplemented with 10% FBS and antibiotics (*penicillin*–*streptomycin*; 10,000 U/mL; final concentration in culture medium was 1%).

#### 3.1.2. MTT Viability Test

The cells were seeded at a density of 1 × 10^4^ in 96-well plates. Following overnight culture, the cells were treated with increasing doses of compounds **1**–**3** (i.e., 10, 25, 50, or 100 μM) and incubated for 24 h. Next, 10 µL of MTT (3-(4,5-dimethylthiazolyl-2)-2,5-diphenyltetrazolium bromide) reagent (Cayman) was added to each well and after 3 h of incubation (37 °C, 5% CO_2_) the medium was aspirated, and the formazan produced in the cells appeared as dark crystals at the bottom of the wells. Subsequently, crystal dissolving solution (Cayman) was added to each well and the optical density (OD) was determined at 570 nm on a plate reader (BIOTEK). Doxorubicin (DOX) was used as a positive control. When hepatotoxicity of test compounds was evaluated, the morphology of the cells was also determined using a bright-field inverted microscope (Nikon).

#### 3.1.3. LDH Viability Test

The cells were seeded at a density of 1 × 10^4^ in 96-well plates. Following overnight culture, the cells were treated with increasing doses of **1**–**3** (i.e., 10, 25, 50, or 100 μM) and incubated for 24 h. Subsequently, 100 µL of cell supernatant was transferred to a new 96-well plate and 100 µl of a reaction solution (Cayman) was added to each well. Cells were incubated for 30 min at 37 °C and absorbance was measured at 490 nm. The percent of cytotoxicity of the test samples was determined according to the following formula: ((experimental value A490) − (spontaneous release))/((maximum release) − (spontaneous release)) × 100%, where spontaneous release is the absorbance origin from the control cells, and maximum release is the absorbance origin from the cells treated with TRITON-X-100.

#### 3.1.4. Data Analysis

Data from the cytotoxicity assays were analyzed using Mann–Whitney U test with GraphPad Prism 4.0 Software (GraphPad Software Inc., San Diego, CA, USA). Values of *p* < 0.05 were considered to be statistically significant.

### 3.2. Metabolic Activation and Trapping Assay

#### 3.2.1. Chemicals and Reagents

Human liver microsomes (HLMs), NADPH-regenerating system components, and reduced glutathione were purchased from Sigma-Aldrich (St Louis, MO, USA).

#### 3.2.2. Human Liver Microsomal Biotransformation and Trapping Assay

The reaction systems (total volume of 250 μL) contained compounds **1**, **2**, or **3** (50 μM), glutathione (1 mM), HLMs (1 mg/mL), NADPH-regenerating system, and 0.1 M potassium phosphate buffer (pH 7.4). After 10 min preincubation at 37 °C, the reaction was started by adding the NADPH-regenerating system (NADP, glucose-6-phosphate, and glucose-6-phosphate dehydrogenase in a potassium phosphate buffer). Incubation was conducted for 60 min at 37 °C in a shaker. Then 70% perchloric acid was used to terminate the reaction. After centrifugation at 10,000 rpm for 10 min, the supernatant was collected and analyzed by UPLC–MS/MS (Waters Corporation, Milford, MA, USA) for direct analysis of glutathione adducts. Control reactions were carried out in the absence of glutathione. All probes were done in duplicate [59].

### 3.3. Mutagenicity and Antimutagenicity Assays

#### 3.3.1. Chemicals and Reagents

The following chemicals and reagents were obtained from Sigma-Aldrich (St Louis, MO, USA): magnesium sulfate, citric acid monohydrate, potassium phosphate dibasic, sodium ammonium phosphate, L-histidine, D-biotin, sodium phosphate dibasic, sodium phosphate monobasic, magnesium chloride, potassium chloride, and 4-nitroquinoline-*N*-oxide (NQNO). Agar, bacto-peptone, and beef extract were purchased from Merck (Darmstadt, Germany). D-Glucose, NaCl, and DMSO were obtained from Chempur (Piekary Śląskie, Poland), and nutrient broth no. 2 from Oxoid (Hampshire, UK). Glycerol and neomycin sulfate were purchased from Pharma Cosmetic (Krakow, Poland).

#### 3.3.2. Bacterial Test Systems

*S. typhimurium* TA100 strain was kindly provided by Dr. K.I. Sugiyama (National Institute of Hygienic Sciences, Tokyo, Japan); *V. harveyi* strains were a gift from Prof. G. Węgrzyn (Department of Molecular Biology, University of Gdańsk, Poland).

#### 3.3.3. Ames Mutagenicity Testing

The Ames assay was conducted by the preincubation method using *S. typhimurium* TA100 strain [60]. Each probe was performed in triplicate; 100 µL of the overnight bacterial culture, 50 µL of the investigated compounds at a concentration of 40 ng/mL, and 500 µL of a buffer solution were preincubated for 30 min at 37 °C and then added to 2 mL of the top agar containing *histidine*/*biotin solution*. The resulting mixture was poured onto minimal glucose plates. After 48 h incubation, the revertants were counted manually and the results were expressed as mutagenic index (MI) (MI = the number of revertant colonies induced in the tested sample/the number of spontaneous revertants in the negative control). The mutagenic potential of a test compound was stated if the mutant frequency was 2.0 or higher [61,62]. The standard mutagen used as a positive control was NQNO. DMSO served as the negative (solvent) control.

#### 3.3.4. *Vibrio harveyi* Mutagenicity Testing

In the experiment, overnight cultures of bacterial strains BB7, BB7M, BB7X, and BB7XM were used. Each culture was centrifuged and 10 µL of *V. harveyi* pellets was placed in 20 mL of BOSS medium. The culture was cultivated at 30 °C until the optical density reached 0.1 at 600 nm (OD_600_). Then, 10 µL of test compound solution (final concentration of 40 ng/mL) was added to the bacterial culture and incubated. Incubation was continued until OD_600_ reached 0.3–0.4. Next, 5 × 10^6^ bacterial cells were spread onto BOSS agar plates with neomycin. The *plates were incubated* at 30 °C for 48 *h* and the number of neomycin-resistant colonies was counted. As a positive control, NQNO was used, whereas DMSO and water served as negative controls [63,64,65]. All the experiments were analyzed in three independent repetitions. Finally, MI was calculated. A compound was considered mutagenic if MI was equal or greater than 2.0 [61,62].

#### 3.3.5. Ames Antimutagenicity Testing

The antimutagenic properties of compounds **1**–**3** were assayed in *S. typhimurium* TA100 strain against NQNO with a modified method of Maron and Ames [60].

Triplicate plates were set up with each test compound concentration and the entire experiment was repeated twice. The inhibition of mutagenicity was expressed as a percentage decrease of reverse mutation, calculated using the following equation: percent inhibition = 100 − ((R_1_/R_2_) × 100), where R_1_ is the number of revertants per plate induced by test compound plus mutagen, and R_2_ is the number of revertants per plate induced by a mutagen alone; 25–40% inhibition was defined as moderate antimutagenicity, 40% or higher inhibition was defined as strong antimutagenicity, and 25% or less inhibition indicated no antimutagenicity [66,67].

#### 3.3.6. *Vibrio harveyi* Antimutagenicity Testing

The antimutagenicity assay was conducted according to the same procedure as used for the mutagenicity assay, except that the standard mutagen was also added to the bacterial cultures incubated with test compounds [65]. The inhibition percentage of mutagenicity was calculated as described above in the Ames antimutagenicity assay procedure.

### 3.4. P-gp ATPase Activity Assay

The changes in the ATPase activity of P-gp were determined by using the P-gp-Glo TM assay system (Promega). Verapamil (VER) was used as a positive control since it inhibits P-gp ATPase activity, which eventually leads to reduced drug efflux. When P-gp ATPase activity is inhibited, ATP accumulates and becomes a substrate for luciferase, with a consequent increase in luminescence. The tested compounds at increasing concentration (10–100 µM) were incubated with 5 mM Mg ATP and 25 µg recombinant human P-gp membranes at 37 °C for 45 min. The luminescence signal was started by adding ATP detection buffer. After incubation at room temperature for 25 min, the samples were read in white 96-well plates on a multi-plate reader (BIOTEK).

## 4. Conclusions

Compounds **1**–**3** were designed and synthesized based on a *trans*-2-aminocyclohexan-1-ol structure. Previous investigations have indicated the anticonvulsant potency of these compounds. Additionally, in vitro metabolism of **1**–**3** via liver microsomes and a microbial model (*Cunninghamella*) was performed. To complement the above results, in the present study the safety profile of both enantiomers and a racemate (**1**–**3**) was evaluated, including their cytotoxicity, mutagenicity, and reactive metabolite formation potential. Moreover, their antimutagenic activity and the effect on P-gp ATPase were examined.

The obtained results revealed that the newly synthesized active chiral *N*-aminoalkyl derivatives of *trans*-2-aminocyclohexan-1-ol may be promising candidates for further research focused on the discovery and development of anticonvulsant agents. All tested compounds demonstrated their promising anticancer properties on the glioblastoma cells U373. Furthermore, the P-gp ATPase assay demonstrated that both enantiomers are potent P-gp inhibitors as they inhibit P-gp ATPase activity. This new application of compounds **1**–**3** is also attractive due to their mutagenic safety, low bioactivation potential, and important antimutagenic activity. These results might be of great potential interest in the design of new antiepileptic compounds.

Finally, our study indicated the similar safety profile of the enantiomeric *N*-aminoalkyl derivatives of *trans*-2-aminocyclohexan-1-ol, although in the previous studies both enantiomers differ in their biotransformation pathways and pharmacological activity.

## Figures and Tables

**Table 1:** Anticonvulsant quantification results (in vivo study) [9].

Compound	Dose (mg/kg)	TD_50_^a^	ED50_(MES)_^b^	PI_(MES)_
**1**	30	66.03 ^#^	38.34 ^#^	1.722 ^#^
	100	66.03 ^#^_scMet_	>90.00 ^#^_scMet_	<1.722 ^#^_scMet_
**2**	30	n.d.	n.d.	n.d.
	100	n.d.	n.d.	n.d.
**3**	30	71.04 ^#^	25.05 ^#^	2.839 ^#^
	100	71.04 ^#^_scMet_	>150 ^#^_scMet_	<0.474 ^#^_scMet_
	300	>250 ^^^	99.25 ^^^	2.519 ^^^
PHT		34.45 ^#^	6.48 ^#^	6.60 ^#^
		>500 ^^^	32.2 ^^^	>22 ^^^
CBZ		47.8 ^#^	9.85 ^#^	4.90 ^#^
		361 ^^^	3.57 ^^^	101 ^^^
VPA		483 ^#^	287 ^#^	1.70 ^#^
		859 ^^^	395 ^^^	2.2 ^^^

^a^ Dose of the compound that produces toxicity in 50% of tested animals; ^b^ dose of the compound that gives protection against seizures toxicity in 50% of tested animals. ^#^ Values for mice after i.p. administration. ^ Values for rats after p.o. administration; n.d.—not determined. Anticonvulsant quantification were determined only for compounds **1** and **3**, which display sufficient antiepileptic activity and low neurotoxicity in the primary evaluations; PHT: phenytoin, BZ: carbamazepine, VPA: valproate.

**Table 2:** Mutagenic activity of tested compounds in the Ames and the *Vibrio harveyi* tests.

Compound	Ames Test		*Vibrio harveyi* Test							
	TA100 ^a^		BB7 ^a^		BB7X ^a^		BB7M ^a^		BB7XM ^a^	
	Mean ± SD	MI ^b^	Mean ± SD	MI ^b^	Mean ± SD	MI ^b^	Mean ± SD	MI ^b^	Mean ± SD	MI ^b^
H_2_O	8 ± 3		19 ± 4		17 ± 6		22 ± 3		9 ± 4	
DMSO	16 ± 5		16 ± 3		13 ± 4		16 ± 2		11 ± 4	
NQNO	33 ± 4	2.1	32 ± 4	2.0	26 ± 2	2.0	32 ± 2	2.0	24 ± 3	2.2
**1**	9 ± 4	0.6	21 ± 3	1.1	16 ± 5	0.9	22 ± 3	1.0	18 ± 2	1.6
**2**	3 ± 1	0.2	23 ± 5	1.2	10 ± 6	0.6	23 ± 3	1.1	10 ± 3	0.9
**3**	6 ± 3	0.4	23 ± 3	1.4	13 ± 4	1.0	27 ± 4	1.7	15 ± 4	1.4

^a^ Number of revertants; NQNO (nitroquinoline-*N*-oxide, 40 ng/mL) was used as a positive control; DMSO and H_2_O were used as a negative control; ^b^ MI (mutagenic index): number of induced revertants/number of spontaneous revertants (positive assay when MI ≥ 2).

**Table 3:** Antimutagenic activity of compounds **1**–**3** in the Ames and the *Vibrio harveyi* tests.

Compound	Ames Test		*Vibrio harveyi* Test							
	TA100 ^a^		BB7 ^a^		BB7X ^a^		BB7M ^a^		BB7XM ^a^	
	Mean ± SD	Inhib. (%) ^b^	Mean ± SD	Inhib. (%) ^b^	Mean ± SD	Inhib. (%) ^b^	Mean ± SD	Inhib. (%) ^b^	Mean ± SD	Inhib. (%) ^b^
H_2_O	5 ± 2		12 ± 2		11 ± 4		16 ± 3		13 ± 4	
DMSO	6 ± 2		14 ± 3		13 ± 3		10 ± 2		15 ± 3	
NQNO	22 ± 4		38 ± 5		29 ± 4		35 ± 3		24 ± 4	
**1**	16 ± 2	(27)	26 ± 3	(32)	20 ± 4	(31)	22 ± 2	(37)	16 ± 2	(35)
**2**	14 ± 3	(38)	23 ± 4	(38)	21 ± 3	(30)	20 ± 4	(41)	14 ± 4	(40)
**3**	13 ± 3	(41)	19 ± 3	(51)	21 ± 4	(29)	22 ± 5	(36)	10 ± 4	(57)

^a^ Number of revertants; NQNO (nitroquinoline-*N*-oxide, 40 ng/mL) was used as a positive control; DMSO and H_2_O were used as a negative control; ^b^ the values in parenthesis are the inhibition rates (%) of mutagenicity; 25–40% inhibition: moderate antigenotoxicity, 40%; or more inhibition: strong antigenotoxicity, 25% or less inhibition: no antigenotoxicity.
